# Don’ts for Consumptives, or the Scientific Management of Pulmonary Tuberculosis

**Published:** 1896-06

**Authors:** 


### BOOK NOTICES.

Don'ts for Consumptives, or The Scientific Management of Pulmonary Tuberculosis. How the Pulmonary Invalid May Make and Maintain a Modern Sanatorium of His Home, with Additional Chapters Descriptive of How Every Consumptive Person May Apply the Forces of Nature to Assist and Hasten Recovery, and also how the Defects of Heredity may be best overcome. By Charles Wilson Ingraham, M. D., Binghamton, N. Y. February, 1896. Printed for the author by *The Call*, Binghamton.

This valuable book consists of thirty-eight chapters, each chapter being preceded by a text which may be called a "Don't." We will give a few of these "don'ts:"

Chapter I. Do not adopt any method for the disposal of expectorated material except that which provides for its absolute destruction by fire.

Chapter III. Do not allow the smallest particle of expectoration to remain upon your sheets, bed-coverings, or any other articles of furniture.

Chapter V. Do not occupy rooms which have been previously occupied by a consumptive person, unless you know positively that the apartments and hallways or walks leading to them have been effectively cleansed and disinfected.

Chapter X. Do not antagonize the adoption and enforcement of any just municipal or State legislation designed to limit or control tubercular contagion.

Chapter XII. Do not neglect systematic chest exercises designed to strengthen the muscles of respiration and to increase the breathing capacity of the lungs.

Chapter XIV. Do not go suddenly into cold or damp air and take a deep, or even an ordinary inhalation.

Chapter XV. Do not allow yourself to breathe through the mouth.

Chapter XXIII. Do not neglect giving the teeth the best of care.

Chapter XXIV. Do not make use of a patent cough syrup.

These "dont's," selected at random, show the range of the book. Each one is followed by a chapter of commentaries, by which the value of the "don't" is made more clear. They are excellent and most useful.

The book ought to be put in the hands of every consumptive, and it would be a good idea for physicians to supply themselves with a number of copies, so that when they have consumptive cases a copy of the book may be handed to the patient, and thus save the physician much talking when directing the medical care of the case.

From this it will be seen that the author has a great belief in the contagiousness of consumption. He also is a firm believer in the ultimate extermination of consumption through carrying out the ideas of Prof. Koch, of Berlin. It may be added that he is an advocate of the corrollary of these ideas, a strict hygiene, and of legislation to enforce this hygiene. This book then sets forth the practical sanitary measures which the patient himself may make use of to bring about recovery and prevent infection of those with whom he is brought in contact.

